# Intravenous immunoglobulin response in new-onset refractory status epilepticus (NORSE) COVID-19 adult patients

**DOI:** 10.1007/s00415-021-10468-y

**Published:** 2021-03-11

**Authors:** Paolo Manganotti, Giovanni Furlanis, Miloš Ajčević, Cristina Moras, Lucia Bonzi, Valentina Pesavento, Alex Buoite Stella

**Affiliations:** 1 Clinical Unit of Neurology, Department of Medicine, Surgery and Health Sciences, Trieste University Hospital-ASUGI, University of Trieste, Strada di Fiume, 447-34149 Trieste, Italy; 2 Department of Engineering and Architecture, University of Trieste, Via Alfonso Valerio, 10, Trieste, Italy; 3 Unit of Internal Medicine, Department of Medicine, Surgery and Health Sciences, Trieste University Hospital-ASUGI, University of Trieste, Strada di Fiume, 447-34149 Trieste, Italy; 4 Rehabilitation Unit, Department of Medicine, Surgery, and Health Sciences, Trieste University Hospital-ASUGI, University of Trieste, Via Giuseppe Lorenzo Gatteri 25/1, Trieste, Italy

**Keywords:** COVID-19, NORSE, Intravenous immunoglobulin, Epilepsy, Encephalitis

## Abstract

Neurological manifestations may be common in COVID-19 patients. They may include several syndromes, such as a suggested autoimmune abnormal response, which may result in encephalitis and new-onset refractory status epilepticus (NORSE). Quickly recognizing such cases and starting the most appropriate therapy is mandatory due to the related rapid worsening and bad outcomes. This case series describes two adult patients admitted to the university hospital and positive to novel coronavirus 2019 (SARS-CoV-2) infection who developed drug-resistant status epilepticus. Both patients underwent early electroencephalography (EEG) assessment, which showed a pathological EEG pattern characterized by general slowing, rhythmic activity and continuous epileptic paroxysmal activity. A suspected autoimmune etiology, potentially triggered by SARS-CoV-2 infection, encouraged a rapid work-up for a possible autoimmune encephalitis diagnosis. Therapeutic approach included the administration of 0.4 g/kg intravenous immunoglobulin, which resulted in a complete resolution of seizures after 5 and after 10 days, respectively, without adverse effects and followed by a normalization of the EEG patterns.

## Introduction

Novel coronavirus disease (COVID-19) is mostly known to affect the respiratory system, potentially leading to severe acute respiratory failure. Yet, most patients with COVID-19 have also shown nonspecific neurological symptoms, such as confusion or headache, and some of them, particularly those with severe COVID-19-related respiratory failure, developed specific neurological manifestations, such as seizure or cerebrovascular events.

New-onset refractory status epilepticus (NORSE) may occur as a consequence of COVID-19. NORSE is a condition defined as the occurrence of refractory status epilepticus in patients without active epilepsy and without a clear acute or active structural, toxic or metabolic cause. The most frequently identified cause of NORSE is autoimmune encephalitis. Indeed, patients with refractory status epilepticus caused by anti N-methyl-D-aspartate receptor (NMDAr) encephalitis without lung involvement were recently reported in COVID-19 patients [1, 2], such as probable autoimmune encephalitis and encephalomyelitis [3]. NMDAr encephalitis represents the most frequent cause of autoimmune encephalitis and it may be triggered by viral infection, particularly Herpes Simplex Virus [4]. Early immune therapy (steroids, intravenous immunoglobulins, and plasma exchange) is recommended for autoimmune encephalitis-related NORSE treatment, since a delayed treatment may contribute to worse outcomes.

The aim of this report is to encourage a rapid diagnostic work-up and implementation of intravenous immunoglobulin (IVIG) therapy in possible COVID-19 autoimmune encephalitis-related NORSE. As such, we described two NORSE patients affected by COVID-19 who successfully responded to the IVIG, thus suggesting the basic autoimmune mechanisms in these COVID-19 epileptic statuses.

## Materials and methods

This case series described two patients admitted to the hospital affected by bilateral pneumonia due to the novel Coronavirus 2019 (SARS-CoV-2) infection from March to December 2020 diagnosed by a positive nasopharyngeal swab test. Due to positivity for COVID-19, the patients were admitted to the COVID-19 protected areas of the University Hospital of Trieste. COVID-19 diagnosis was confirmed through nasopharyngeal swab testing. COVID-19 management included steroids (dexamethasone 6 mg/die for 10 days) to treat respiratory insufficiency, venous thromboembolism prophylaxis (enoxaparin 4000 IU), initial broad-spectrum antibiotics followed by specific antibiotics according to the antibiogram, artificial ventilation (case 1: orotracheal intubation; case 2: non-invasive ventilation; followed by, both cases: progressive low-flow oxygen therapy). None of the patients was in prone position. The patients presented (one at the admission and one after 11 days of hospitalization) clinical seizures or reduced vigilance and altered mental status, suggestive of a diagnosis of status epilepticus (SE). All the patients received neurological examination at symptoms development, electroencephalography (EEG), routine blood chemistry analyses, and a panel of diagnostic testing, including neuroimaging and biomarkers. Cerebrospinal fluid (CSF) was collected and processed for standard analyses including pressure, cell count, proteins, and glucose. CSF culture and polymerase chain reaction (PCR) for possible organisms, such as bacteria, Mycobacterium tuberculosis, fungi, Herpes viruses, Enteroviruses, Japanese B virus, and Dengue viruses was performed, including analysis for SARS-CoV-2. Serum and CSF were tested for onconeural antibody, as antiamphiphysin, antiCV2, antiMa2/TA, antiRI, antiYo, antiHu, antirecoverin, antiSox1, antitin, antiZic4, antiGAD65/67 (Anti-Glutamate Decarboxylase), antiTr, and antineuronal surface antigens antibodies, as antiNMDAr (*N*-methyl-d-aspartate receptor), antiVGKC (voltage gated potassium channel) complex LGI1 (leucine-rich glioma inactivated 1) and CASPR2 (Contactin-associated protein-like 2), antiAMPA1r (Anti-α-amino-3-hydroxy-5-methyl-4-isoxazolepropionic acid), antiAMPA2r, antiGABABr (Gamma-aminobutyric acid), antiDPPX (dipeptidyl-peptidase-like protein 6). Due to the lack of response to the antiepileptic drugs, all the patients were compatible with a possible autoimmune encephalitis-related NORSE diagnosis and they were treated with IVIG.

### EEG acquisition and analysis

Thirteen channel 20-min standard clinical surface EEG was acquired by Be Plus PRO amplifier (EB NEURO, Florence, Italy) and 13 Ag/AgCl electrodes (F7, F3, F4, F8, C3, Cz, C4, T5, P3, P4, T6, O1, O2) placed according the standard 10–20 System. All electrode impedances were kept below 5 kΩ, and sampling rate was set to 256 Hz. EEG signals were filtered by second order band-pass Butterworth filter with 0.1–30 Hz cut-off frequencies. Brain oscillatory activities were assessed by qualitative visual inspection of EEG tracings by two experienced neurologists (P.M. and G.F.) to identify epileptiform patterns and altered EEG rhythms. Furthermore, power spectral density (PSD) was estimated on 120 s segments using Welch’s periodogram and absolute power for each of spectral band (*δ*: 1–4 Hz; *θ*: 4–8 Hz; *α*: 8–13 Hz; *β*: 13–30 Hz) was calculated and then normalized with a total power across the 1–30 Hz range to obtain relative powers. The physician and the technician wore personal protective equipment (PPE), including appropriate masks, face shields, gowns and gloves according to the American Association of Clinical Neurophysiology guidelines published on its official website (https://www.acns.org/practice/covid-19-resources).

## Results

### Case 1

**Fig. 1:**
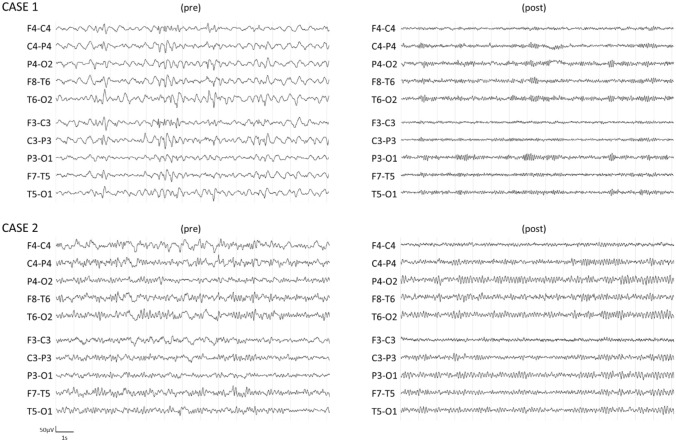
EEG raw data before IVIG therapy (pre)—left panel and after IVIG therapy (post)—right panel. EEG spectral analysis showed EEG relative powers indices pre—post modification; Case 1—pre: *δ* = 0.79, *θ* = 0.16, *α* = 0.03, *β* = 0.02; Case 1—post: *δ* = 0.17, *θ* = 0.15, *α* = 0.48, *β* = 0.20; Case 2—pre: *δ* = 0.55, *θ* = 0.31, *α* = 0.09, *β* = 0.05; Case 2—post: *δ* = 0.16, *θ* = 0.32, *α* = 0.41, *β* = 0.11

A 37-year-old male, with an unremarkable medical history, was admitted to the Intensive Care Unit (ICU), due to convulsive status epilepticus. The nasopharyngeal swab test was found positive for SARS-CoV-2, while serum analysis, head CT scan and toxicological examination were negative. First line treatment and intravenous Levetiracetam infusion (3000 mg/24 h iv) were immediately started but led to no clinical improvement. EEG was performed within the first 24 h, revealing generalized SE (Fig. 1). Additional antiepileptic treatments including Valproic Acid (3000 mg/24 h iv), Phenytoin (18 mg/kg iv) and Lacosamide (400 mg iv in 24 h, increase to 600 mg in 24 h) were progressively administered after following examinations and concomitant to Propofol infusion without significant improvement. Subsequent EEG showed a generalized delta slowing; consequently, continuous infusion of Midazolam was added to Propofol to achieve burst suppression. After 24 h, Propofol was progressively reduced, but the patient suddenly developed generalized myoclonic jerks of axial muscles and face. Continuous EEG monitoring showed persistent generalized epileptic discharges compatible with non-convulsive status epilepticus (NCSE). Early diagnostic work-up was performed as follows: contrast-enhanced Magnetic Resonance Imaging (MRI)—negative results; serum HIV, VDRL and hepatitis virus panel—negative; CSF analysis—CSF protein concentration was 56.7 mg/dL, glucose 66.1 mg/dL, 1 mononuclear white blood cell, and CSF culture and PCR yielded negative results; serum and CSF onconeural antibody and antineuronal surface antigens antibody were found positive for anti amphiphysin antibody. Contrast-enhanced whole-body CT and testicular ultrasound suggested the absence of any neoplastic process. Given the lack of response to multiple antiepileptic drugs and third-line anesthetics drugs, a diagnosis of possible autoimmune encephalitis-related NORSE was done. IVIG therapy was initiated at 0.4 g/kg for 5 days. The EEG showed a dramatic improvement on the fifth day of IVIG infusion, with complete clinical recovery without seizures and a complete normalization of EEG (Fig. 1). The patient was awake and without respiratory support. No side effects were reported for the use of intravenous immunoglobulin therapy. After a negative whole-body PET-CT, the neoplastic origin was excluded and the patient was dismissed from the hospital without severe cognitive defects and with a partial decrease of antiepileptic drugs.

### Case 2

A 71-year-old male, with a history of arterial hypertension, was admitted to the infectious disease unit, due to severe respiratory failure symptoms compatible with COVID-19 bilateral pneumonia. The nasopharyngeal swab test was found positive for SARS-CoV-2 and the patient was treated with non-invasive ventilation due to respiratory distress. After 11 days, the patient became lethargic and showed negative upper limbs myoclonus and random, involuntary, and rapid vertical eye movements. Kidney and liver function biomarkers, serum electrolytes and ammonia were normal, head CT scan and toxicological examination resulted negative. Clinical evaluation (myoclonic positive and negative jerks, and altered mental status) was compatible with NCSE diagnosis, while EEG revealed generalized epileptic discharges with bilateral frontal high amplitude delta waves (Fig. 1). At first, intravenous Valproic Acid (2000 mg/24 h iv) and secondarily Levetiracetam infusion (3000 mg/24 h iv) were started but led to no clinical nor EEG improvement. Early diagnostic work-up was performed as follows: contrast-enhanced MRI—negative results; serum VDRL and hepatitis virus panel—negative; CSF analysis—CSF protein concentration was 53.9 mg/dL, glucose 104 mg/dL, 1 mononuclear white blood cell, CSF culture and PCR yielded negative results, as well as serum and CSF onconeural antibody and antineuronal surface antigens antibody. Given the lack of response to multiple antiepileptic drugs, a diagnosis of possible seronegative autoimmune encephalitis-related NORSE was done. IVIG therapy was initiated at 0.4 g/kg for 5 days. After the first 5-day cycle, due to the scarce response (remission of myoclonic jerks and vigilance partial improvement), a second 5-day IVIG cycle was started with a progressive improvement of both clinical and EEG features (Fig. 1) without side effects. The patient was dismissed from the hospital without severe cognitive defects and with a partial decrease of antiepileptic drugs.

## Discussion

This report fosters the implementation of a rapid diagnostic work-up and use of IVIG as a promising therapy to safely and successfully treat possible autoimmune encephalitis-related NORSE triggered by SARS-CoV-2 infection. A rapid diagnosis and appropriate treatment are essential to avoid irreversible sequelae of autoimmune encephalitis-related NORSE. Neurological features may be common in COVID-19 patients, as reported in different studies, and some authors suggest to suspect SARS-CoV-2 infection when seeing patients with neurologic manifestations during the pandemic period [5, 6]. Indeed, evidence suggests a direct neuronal injury, as shown by increased serum neurofilament light chain (sNfL) levels, in critically ill COVID-19 patients compared to critically ill non-COVID-19 patients [7]. Although the exact pathophysiological mechanisms underlying the development of neurological syndromes are not completely appreciated, some explanatory mechanisms are currently debated, such as (1) a systemic inflammatory response, (2) a prothrombotic state, and (3) direct viral invasion [8]. Besides, the reported cases suggest that SARS-CoV-2 infection could trigger autoimmune responses, with a Central Nervous System (CNS) involvement. From a historical perspective, one century ago the “pandemic flu” was followed by an outbreak of encephalitis [9]. The negativity of PCR for other viruses on cerebrospinal fluid and the temporal association between SARS-CoV-2 infection and neurological symptoms suggests a possible causative role of COVID-19 in the development of the autoimmune response, thus resulting in NORSE.

Recently, some patients with autoimmune encephalitis associated with COVID-19 have been reported, in which irritability, confusion, drowsiness, and new-onset epilepsy account for the main symptoms at onset [1–3]. In our study, we reported two cases of encephalitis associated with NORSE rapidly assessed with EEG. The early EEG testing contributes to the rapid recognition of electric abnormalities, which may be common in COVID-19 patients [10, 11] also due to secondary causes, such as cerebrovascular events, electrolytes imbalance, oxidative stress, mitochondrial dysfunction, hypoxia, and prolonged inactivity [12–15]. Despite the fact that continuous EEG monitoring should be preferred, this study could rely only on daily spot EEG measurements due to the complexity of evaluation and testing in a COVID-19 area. Disturbances of background activity have been reported in most COVID-19 patients with seizures, such as generalized and focal slowing and a high level of epileptiform abnormalities and rhythmic or periodic discharges [10–12]. Additionally, the finding of slightly increased CSF protein concentration, without significant cells’ increase, supported the hypothesis of autoimmune mechanisms, although it may also reflect other non-autoimmune neurological diseases.

Our cases highlighted the importance of suspecting an autoimmune cause in NORSE patients positive to SARS-CoV-2 after excluding structural, infectious, metabolic, and toxic etiologies, and that IVIG treatment should be rapidly started to promote symptoms remission, while a complete autoimmune work-up needs to be set up as early as possible to confirm the diagnosis. Although the use of IVIG in NORSE is not supported by conclusive findings [16], the use of such therapy in possible encephalitis-related NORSE in COVID-19 patients suggested favorable results also in large cohorts of patients [3] and in COVID-19 patients affected by other autoimmune peripheral neurological syndromes (e.g., Guillain–Barré and Miller Fisher) [17–20]. The rapid and remarkable improvement of both our patients from resistant status epilepticus not responsive to different antiepileptic drugs is in line with the above-mentioned studies.

## Conclusions

Autoimmune encephalitis may be a consequence of COVID-19 and it may result in NORSE. Although a causative relationship cannot be stated due to the retrospective nature of our study, initiating immunotherapy in case of suspected autoimmune etiology is desirable and a rapid diagnostic process should be encouraged (including EEG monitoring). Our findings support the use of IVIG therapy in NORSE COVID-19 patients with suspected autoimmune encephalitis to prevent negative outcomes, due to the time-dependent course of the disease.
